# Origin of Strong Liquors

**Published:** 1889-05

**Authors:** 


### ORIGIN OF STRONG LIQUORS.

Strong liquors are a modern invention. The ancients knew of nothing more powerful than lightly fermented wines, and have left warnings enough of the abuse of them. Alcohol was not discovered till the seventh century, although an older story exists of a monk, Marcus, who collected and condensed in wool the steam of heated white wine, and then pressed out from the wool a balsam which he applied to the wounds of those who fell at the siege of Rheims, in the reign of Clovis I. He also mixed this balsam with honey and produced a cordial which brought the moribund back to life. Clovis, however, did not wait for the approach of death before claiming his share of the cordial. According to Dr. Stanford Chaelle, the distillation of spirit from wine was not discovered until the twelfth century, and spirits did not come into common use until the fifteenth, sixteenth and seventeenth centuries. Prof. Arnoldus Villanova, in the fourteenth century, made a panacea of the water of life, which gave sweet breath and fortified the memory, besides being good for sore eyes, the toothache and the gout, and having other wonderful properties.

Distilled spirits came into use in London in 1450, and had to be prohibited in 1494. Michael Savonarola produced a treatise on the making of the water of life, in the fifteenth century, which became a standard authority on the subject, and was followed by the work of Matthioli of Sienna. These books gave the start to brandy-making in Italy, whence the trade extended to France. About 1520 the Irish usquebaugh began to acquire reputation in England. Before 1601 "brand-wine" had begun to be distilled in the Low Countries from apples, pears and malt; and in that year an ordinance was passed at Tournay forbidding the sale of the liquor except by apothecaries, "partly because of the dearness of corn and partly because of the drunkenness which this cheap brand-wine caused, to the great prejudice not alone of home and lives, but to the extreme danger of the souls of its drinkers, many of whom had died without confession."—*Druggists' Circular.*

